# Breakthrough SARS-CoV-2 infections in double and triple vaccinated adults and single dose vaccine effectiveness among children in Autumn 2021 in England: REACT-1 study

**DOI:** 10.1016/j.eclinm.2022.101419

**Published:** 2022-05-06

**Authors:** Marc Chadeau-Hyam, Oliver Eales, Barbara Bodinier, Haowei Wang, David Haw, Matthew Whitaker, Joshua Elliott, Caroline E. Walters, Jakob Jonnerby, Christina Atchison, Peter J. Diggle, Andrew J. Page, Deborah Ashby, Wendy Barclay, Graham Taylor, Graham Cooke, Helen Ward, Ara Darzi, Christl A. Donnelly, Paul Elliott

**Affiliations:** a School of Public Health, Imperial College London, Norfolk Place, London W2 1PG, UK; b MRC Centre for Environment and Health, School of Public Health, Imperial College London, UK; c MRC Centre for Global infectious Disease Analysis and Jameel Institute, Imperial College London, UK; d Department of Infectious Disease, Imperial College London, UK; e Imperial College Healthcare NHS Trust, UK; f National Heart and Lung Institute, Imperial College Healthcare NHS Trust, UK; g CHICAS, Lancaster Medical School, UK and Health Data Research, Lancaster University, UK; h Quadram Institute, Norwich, UK; i National Institute for Health Research Imperial Biomedical Research Centre, UK; j Institute of Global Health Innovation, Imperial College London, UK; k Department of Statistics, University of Oxford, UK; l Health Data Research (HDR) UK, Imperial College London, UK; m UK Dementia Research Institute, Imperial College London, UK

**Keywords:** SARS-CoV-2 prevalence, School-aged children, Vaccine effectiveness, Children vaccination, Booster dose

## Abstract

**Background:**

Prevalence of SARS-CoV-2 infection with Delta variant was increasing in England in late summer 2021 among children aged 5 to 17 years, and adults who had received two vaccine doses. In September 2021, a third (booster) dose was offered to vaccinated adults aged 50 years and over, vulnerable adults and healthcare/care-home workers, and a single vaccine dose already offered to 16 and 17 year-olds was extended to children aged 12 to 15 years.

**Methods:**

SARS-CoV-2 community prevalence in England was available from self-administered throat and nose swabs using reverse transcriptase polymerase chain reaction (RT-PCR) in round 13 (24 June to 12 July 2021, *N* = 98,233), round 14 (9 to 27 September 2021, *N* = 100,527) and round 15 (19 October to 5 November 2021, *N* = 100,112) from the REACT-1 study randomised community surveys. Linking to National Health Service (NHS) vaccination data for consenting participants, we estimated vaccine effectiveness in children aged 12 to 17 years and compared swab-positivity rates in adults who received a third dose with those who received two doses.

**Findings:**

Weighted SARS-CoV-2 prevalence was 1.57% (1.48%, 1.66%) in round 15 compared with 0.83% (0.76%, 0.89%) in round 14, and the previously observed link between infections and hospitalisations and deaths had weakened. Vaccine effectiveness against infection in children aged 12 to 17 years was estimated (round 15) at 64.0% (50.9%, 70.6%) and 67.7% (53.8%, 77.5%) for symptomatic infections. Adults who received a third vaccine dose were less likely to test positive compared to those who received two doses, with adjusted OR of 0.36 (0.25, 0.53).

**Interpretation:**

Vaccination of children aged 12 to 17 years and third (booster) doses in adults were effective at reducing infection risk. High rates of vaccination, including booster doses, are a key part of the strategy to reduce infection rates in the community.

**Funding:**

Department of Health and Social Care, England.

**Research in context**

**Evidence before this study**

A search of PubMed using title or abstract terms “vaccine effectiveness”, “SARS-CoV-2” and “Delta” without language or other restrictions, identified 49 results (with no duplicates). Outcomes varied between studies including all infections, symptomatic infections, high viral loads and severe cases of COVID-19 (including deaths), and some studies focussed on particularly vulnerable or highly exposed populations such as care home residents and frontline workers.

**Added value of this study**

We analysed data from self-administered throat and nose swabs collected by a randomly selected sample of residents of England, aged 5 years and older of the REal-time Assessment of Community Transmission-1 (REACT-1) study. We estimated weighted prevalence in England in mid-October to early November that was approximately twice that estimated in September. Using linked vaccination data and following the rollout of single vaccine doses to children aged 12 to 17 years, we estimated in round 15 64.0% (50.9%, 70.6%) vaccine effectiveness against any infection and 67.7% (53.8%, 77.5%) against symptomatic infections. Adults who received a third vaccine dose were approximately three times less likely to test positive compared to eligible adults who had only received two vaccine doses.

**Implications of all the available evidence**

Population surveys provide a robust basis for the characterisation of transmission dynamics nationally and within sub-populations, such as school-aged children. The population-level impacts of the SARS-CoV-2 vaccination programme are visible in the reduced incidence of both hospital admissions and COVD-19-related deaths. The findings of clear benefits to 12 to 17 year olds of single vaccine doses and to adults of booster doses demonstrate the ability of vaccines developed against the original SARS-CoV-2 virus to protect against Delta variants.

Alt-text: Unlabelled box

## Introduction

Since May 2020, the REal-time Assessment of Community Transmission-1 (REACT-1) study^1^, ^2^, ^3^ has been tracking the spread of the SARS-CoV-2 virus in England approximately monthly. While the first REACT-1 survey (May 2020) captured the decline of the first wave in England, subsequent surveys have characterised the second and third waves. The third wave of infection began coincident with the rapid spread of the Delta variant of SARS-CoV-2 from May 2021 and has continued through to December 2021.

The national vaccination programme against COVID-19 in England (along with the other countries in the United Kingdom [UK]) began in December 2020, with those at highest risk of exposure (healthcare workers) and people at highest risk of serious outcomes (those who are older and/or with particular health conditions) being offered the first doses mainly using ChAdOx1 nCoV-19 (Oxford-AstraZeneca) vaccine. Over time the groups being offered vaccination were extended to include all adults, then children aged 16 and 17 years, and subsequently children aged 12 to 15 years. Although vaccines are highly successful at reducing hospitalisation and death associated with SARS-CoV-2 infection,^4^^,^^5^ individuals who have received two doses of vaccine have a lower, but still appreciable, risk of becoming infected with the Delta variant in the home compared with people who are unvaccinated.^6^

The national vaccine programme was extended to school-aged children aged 12 to 15 years (single dose) in September 2021, while third (booster) doses, at least six months (or at least five months for the most vulnerable^7^) following the second dose, were offered to health and social care workers, all those aged 50 years and over as well as younger people at risk. Both children single vaccine dose and adults third (booster) dose used the BNT162b2 (Pfizer–BioNTech) or the mRNA-1273 (Moderna) vaccine. By mid-December 2021, over 23,500,000 people in the UK had received a booster dose. Subsequently, it has been announced that the offer of booster doses would be extended to all adults^8^ and that a second dose of the Pfizer-BioNTech vaccine would be offered to 16 and 17 year olds throughout the UK.^9^

A study in Israel compared infection prevalence and severe illness among adults aged 60 years and older who had received two vaccine doses with those who had also received a booster dose. At least 12 days after the booster dose, the rate of confirmed infections was 11.3 (95% CI: 10.4, 12.3) times lower and the rate of severe illness was 19.5 (95% CI: 12.9, 29.5) times lower in those who had received the booster compared to those who had received two doses of vaccine.^10^ These results are consistent with a detailed immunological study of 23 adults that demonstrated that a third dose of Pfizer-BioNTech increased SARS-CoV-2 neutralization administered 7 to 9 months after the second dose.^11^

We used data from round 13 (24 June to 12 July 2021), round 14 (9 to 27 September 2021) and round 15 (19 October to 5 November 2021) of the REACT-1 study each including a representative sample of the resident population with RT-PCR test results, hence providing a population-based description of the COVID-19 epidemiological situation in England during these periods. Individual data were linked to National Health Service (NHS) vaccination data in consenting participants to investigate vaccine effectiveness in children aged 12 to 17 years and to compare swab-positivity rates in adults having received a third dose with those having received two doses. The study was carried out against a backdrop of dominance of the Delta variant in England at that time.

## Methods

### Study population

The REACT-1 study methods are available elsewhere.^1^ Data collection took place each month over a two- to three-week period since May 2020, except for December 2020 and August 2021. The present report relates to data on 100,112 individuals obtained during round 15, which was carried out from 19 October to 5 November 2021 (including a further 93 people who took part from 6 to 8 November 2021); 100,527 individuals in round 14 (9 to 27 September 2021), and 98,233 individuals in round 13 (24 June to 12 July 2021). At each round, a random cross-section of the population of England (ages 5 years and over) was invited into the study using as the sampling frame the NHS list of patients registered with a general practitioner in England held by NHS Digital. Up to round 11 (15 April to 3 May 2021) sampling was designed to achieve approximately equal numbers of participants for each lower-tier local authority (LTLA, *n*=315 in England), but from round 12 (20 May to 7 June 2021), we switched to inviting a random sample of the population in proportion to population size at LTLA level. While this increased the numbers sampled in areas with higher population density and reduced the numbers in more sparsely populated areas, overall prevalence estimates should have been relatively unaffected since weighting is applied to provide representative estimates for England as a whole. Up until round 13 (24 June to 12 July 2021) we obtained dry swabs collected by courier from the participant's home with samples sent to the laboratory on a cold chain. From round 14 we switched to ‘wet’ (saline) swabs, which were then randomly assigned to be picked up by courier (without cold chain) or sent by priority post.^12^ Since there was no difference detected between the two methods, in round 15 swabs were only returned using the priority postal service.

### RT-PCR testing

Participants were sent written and video instructions and asked to obtain a self-administered throat and nose swab at home (or their parent/guardian was asked to administer the swab for children aged 12 years and under). Swabs were then sent for reverse transcriptase polymerase chain reaction (RT-PCR) testing for SARS-CoV-2, with a positive test being recorded if both N gene and E gene targets were detected or if N gene was detected with cycle threshold (Ct) value below 37.

### Demographic and questionnaire and vaccination data

We obtained information on age, sex and residential location from the NHS register, and additional information on ethnicity, household size, occupation, past medical history, potential contact with a COVID-19 case, symptoms and other variables via registration and through an online or telephone questionnaire.^13^ A total of 259,955 (86.9%) participants from rounds 13 to 15 consented to link their REACT-1 profile to vaccination data from their NHS record; these included vaccination status, vaccination date and vaccine type.

### Viral genome sequencing

We sent samples that tested positive (with N gene Ct values < 32 and sufficient volume) to the Quadram Institute, Norwich, UK, for viral genome sequencing. The ARTIC protocol^14^ (version 4) was used for viral RNA amplification and CoronaHiT for preparation of sequencing libraries.^15^ Sequencing data were analysed using the ARTIC bioinformatic pipeline^16^ with lineages assigned using PangoLEARN (version 2021-11-4).^17^

### Statistical analyses

We used R software for the statistical analyses.^18^ We calculated unweighted prevalence of swab-positivity as the proportion testing positive on RT-PCR. We then used rim weighting^19^ to provide estimates of prevalence that were weighted to be representative of the population of England as a whole. We used an exponential model of growth or decay to investigate temporal trends in swab-positivity, assuming that the weighted numbers of positive samples out of the weighted total number of samples per day arose from a binomial distribution. Swabs were assigned to day of swabbing where reported or to day of first scan of the sample by the Post Office otherwise (samples were excluded from the temporal analyses when neither swab date nor scan were recorded). We estimated posterior credible intervals using a bivariate No-U-Turn Sampler with a uniform prior distribution for the probability of swab-positivity on the first day of swabbing and the growth rate.^20^ We estimated the reproduction number R as $R={\left(1+\frac{r}{\beta }\right)}^{n}$ assuming a gamma-distributed generation time^21^ with shape parameter *n*=2.29 and rate parameter *β* = 0.36 (corresponding to a mean generation time of 6.29 days).^22^

We fit a Bayesian penalised-spline (P-spline) model^23^ to the daily data using a No-U-Turn Sampler in logit space to visualise trends in swab-positivity over time. The data were partitioned into approximately 5-day sections by regularly spaced knots, adding further knots beyond the study period to minimise edge effects. Basis splines (b-splines) were defined over the system of knots and the P-spline model consisted of a linear combination of these b-splines. We assumed a second-order random-walk prior for the coefficients of the b-splines, $b_{i}=2b_{i-1}-b_{i-2}+u_{i}$ where $u_{i}∼N\left(0,\rho \right)$. The smoothing parameter ρ was assumed to have a non-informative inverse-gamma prior, ρ∼IG(0.001,0.001). We also fit P-splines separately to three broad age groups (17 years and under, 18 to 54 years, 55 years and over) using a smoothing parameter obtained from the model fit to all data. We then examined the link between swab-positivity data in REACT-1 and publicly available hospitalisations and COVID-19 mortality data (deaths within 28 days of a positive test). First, we fit an analogous P-spline model to both sets of publicly available data assuming a negative-binomial likelihood, with an extra overdispersion parameter that was assumed to have a non-informative constant prior distribution. We then selected a random sample (*N*=1000) from the posterior distributions of the P-spline model fits and fit a simple two-parameter model to the first seven rounds of REACT-1 daily swab-positivity data (seven rounds were selected to represent the pre-vaccination period). The two parameters were a discrete time-lag between swab-positivity and hospitalisation/death time series (1000 time series) and a population-adjusted scaling factor that corresponds to the probability of those testing swab-positive on day i being hospitalised/dying on day i + τ, where τ is the time-lag parameter.

We estimated vaccine effectiveness against infection among children ages 12 to 17 years by combining data from round 13 to 15 (to increase statistical power) and for round 15 alone. We used data linked (with consent) to the national COVID-19 vaccination programme to obtain information on who had received a vaccination. Using the dates from the data linkage, a child was considered to have been vaccinated (one dose) 14 days after administration of the vaccine (being considered unvaccinated before then). We estimated vaccine effectiveness as 1 - odds ratio (OR), where we estimated OR from a logistic regression model comparing swab positivity among vaccinated and unvaccinated individuals, with adjustment for round, age and sex, and Index of Multiple Deprivation (IMD), region and ethnicity. We also estimated adjusted odds of infection comparing adults who had received three doses of vaccine with those who had received two doses, using the same adjustment and including a lag period of 14 days post-vaccination as above.

### Ethics

We obtained research ethics approval from the South Central-Berkshire B Research Ethics Committee (IRAS ID: 283787).

### Role of the funding source

The funders had no role in the design and conduct of the study; collection, management, analysis, and interpretation of the data; and preparation, review of this manuscript. PE, MC-H, CAD had full access to the data and take responsibility for the integrity of the data and the accuracy of the data analysis and for the decision to submit for publication.

## Results

### COVID-19 epidemic in England in Autumn 2021

**Figure 1:**
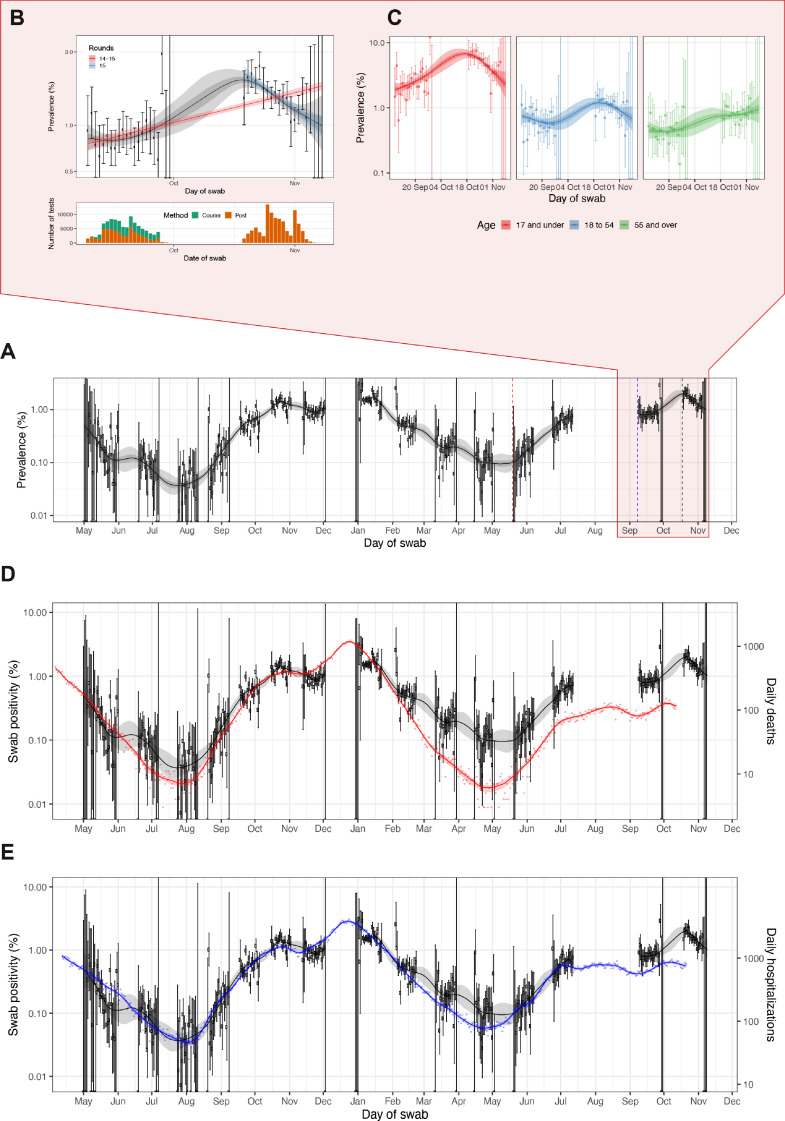
Dynamics of the prevalence of SARS-CoV-2 swab positivity in England. (A) Daily weighted swab-positivity for all 15 rounds of the REACT-1 study (black points with 95% confidence intervals, left-hand y-axis) with P-spline estimates for swab-positivity (solid black line, shaded area is 95% credible interval). Changes in testing procedures are identified by vertical dashed lines. Geographic sampling procedure changed for rounds 12 onwards (red line), round 14 had half of respondents’ swab tests collected by courier and the other half post their swab test (blue line) and for round 15 all respondents posted their swab test (green line)**. (B)** Comparison of an exponential model fit to round 14-15 (red) and round 15 only (blue) and a P-spline model fit to all rounds of REACT-1. Shaded red and blue regions show the 95% posterior credible intervals for the exponential model, and the shaded grey region shows 50% (dark grey) and 95% (light grey) posterior credible interval for the P-spline model. Results are presented for each day (X axis) of sampling for round 14 and round 15 and the prevalence of infection is shown (Y axis) on a log scale. Weighted observations (black dots) and 95% confidence intervals (vertical lines) are also shown. Number of samples processed per day during round 14 and round 15. In round 14 the samples shipped by post are represented in orange, and those shipped by courier, in green. **(C)** Similar comparison of P-spline models fit to all rounds of REACT-1 for those aged 17 years and under (red), those aged 18 to 54 years inclusive (blue) and those aged 55 years and over (green). **(D)** Daily deaths in England (red points, right-hand y-axis) and P-spline model estimates for expected daily deaths in England (solid red line, shaded area is 95% credible interval, right-hand y-axis). Daily deaths have been shifted by 25 (25, 26) days backwards in time along the x-axis. The daily deaths (right-hand) y-axis has been scaled using the best-fit population adjusted scaling parameter 0.060 (0.058, 0.062). **(E)** Daily hospitalisations in England (blue points, right-hand y-axis) and P-spline model estimates for expected daily hospitalisations in England (solid blue line, shaded area is 95% credible interval, right-hand y-axis). Daily hospitalisations have been shifted by 19 (18, 20) days backwards in time along the x-axis. The daily hospitalisations (right-hand) y-axis has been scaled using the best-fit population adjusted scaling parameter 0.238 (0.230, 0.246).

**Figure 2:**
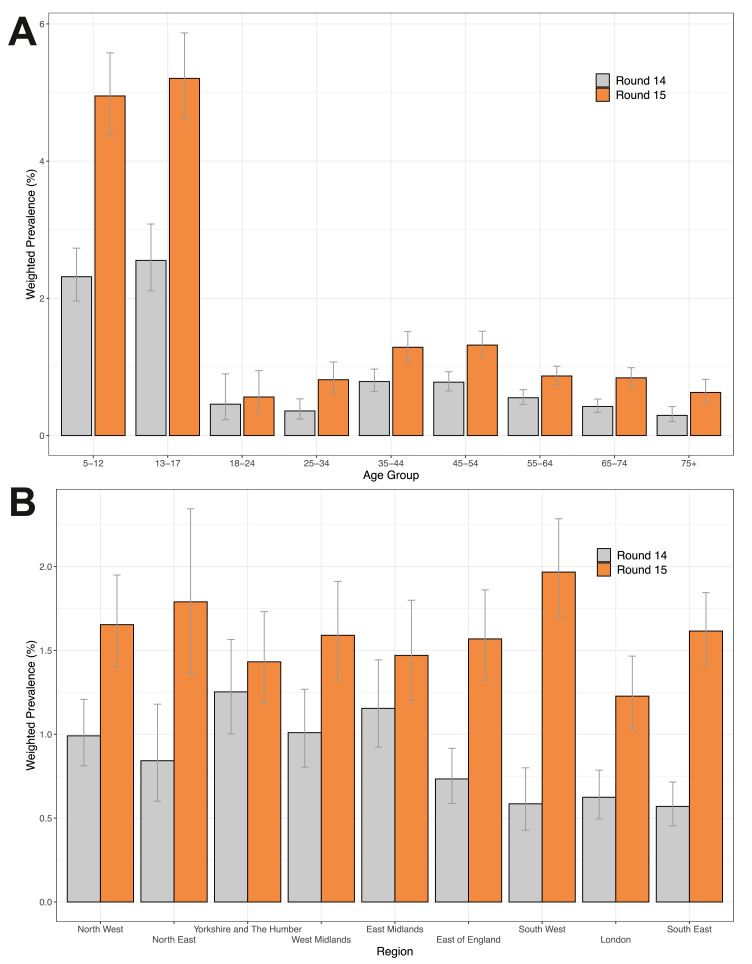
Weighted prevalence of SARS-CoV-2 swab-positivity by age group (A) and region (B). Estimates are presented for round 14 (9 to 27 September 2021) and round 15 (19 October to 5 November 2021). Bars show the prevalence point estimates (grey for round 14 and orange for round 15), and the vertical lines represent the 95% confidence intervals.

A total of 859,184 participants were invited to participate in round 15, of whom 100,112 (11.6%) registered and provided a swab with a valid result from RT-PCR (Supplementary Table 1). Of these 1399 swabs were positive yielding a weighted prevalence of 1.57% (1.48%, 1.66%), nearly two-fold higher than that estimated in round 14 at 0.83% (0.76%, 0.89%) (Figure 1-A, B). We observed the highest weighted prevalence in round 15 in those aged 13 to 17 years at 5.21% (4.61%, 5.87%) and those aged 5 to 12 years at 4.95% (4.39%, 5.58%) (Supplementary Table 2, Figure 2-A). We estimated an average reproduction number across rounds 14 and 15 of *R*=1.09 (1.08, 1.11) with posterior probability that *R*>1 above 0.99 (Supplementary Table 3). The highest weighted prevalence in round 15 by region was observed in South West at 1.97% (1.69%, 2.29%) increasing more than three-fold from round 14 at 0.59% (0.43%, 0.80%) (Supplementary Table 2-A, Figure 2-B). At LTLA level, the ten highest smoothed estimates of prevalence based on a nearest neighbour method were all found in parts of the South West (Supplementary Figure 1-A and B). Sequencing of the positive samples identified 841 lineages, which were all Delta or Delta sub-lineage variants (Supplementary Table 4).

Flexible P-spline models fit to data from all rounds of REACT-1 (Figure 1-A) showed an increasing weighted prevalence of swab-positivity from round 14 to round 15 followed by a fall during round 15 (Figure 1-B). We estimated that the peak in weighted prevalence was reached on around 20 to 21 October (with 95% credible intervals ranging from 15 to 23 October) followed by a fall (Supplementary Figure 2). Similar trends of rising prevalence between round 14 and round 15 followed by a fall within round 15 were observed for children aged 17 years and under and adults aged 18 to 54 years but a fall was not observed at ages 55 years and over (Figure 1-C). Likewise, our exponential model fit to data from round 15 indicated a fall in weighted prevalence during October 2021 with *R*=0.76 (0.70, 0.83) and posterior probability that *R*>1 lower than 0.01 (Supplementary Table 3). Our estimates did not exclude an upturn in the last days of round 15 (from 2 November onwards) based on fewer observations in the first week of November compared to the previous days (Figure 2-B).

We estimated shifting and scaling parameters to overlay daily COVID-19 deaths (deaths within 28 days of a positive test, Figure 2-D) and, separately, daily hospitalisations (Figure 2-E) onto the daily percentage of people who tested swab-positive in REACT-1. We found that the best-fitting time-lag between swab-positivity and hospital admissions was 19 (18, 20) days and it was 25 (25, 26) days for COVID-19 deaths. The best-fitting population adjusted scaling parameter, a measure of the percentage of people swab-positive who will be in hospital or die after the estimated time lag was 0.24% (0.23%, 0.25%) for hospitalisations and 0.060% (0.058%, 0.062%) for deaths. Fewer hospitalisations and COVID-19 deaths occurred from February 2021 after roll-out of the vaccination programme compared to those expected based on the REACT-1 prevalence data, except for hospitalisations during June and July 2021 which coincided with the period during which the Delta variant outcompeted and replaced other strains.

### Vaccination uptake by age, effect of a single vaccine dose in children and of a third vaccine dose in adults

**Figure 3:**
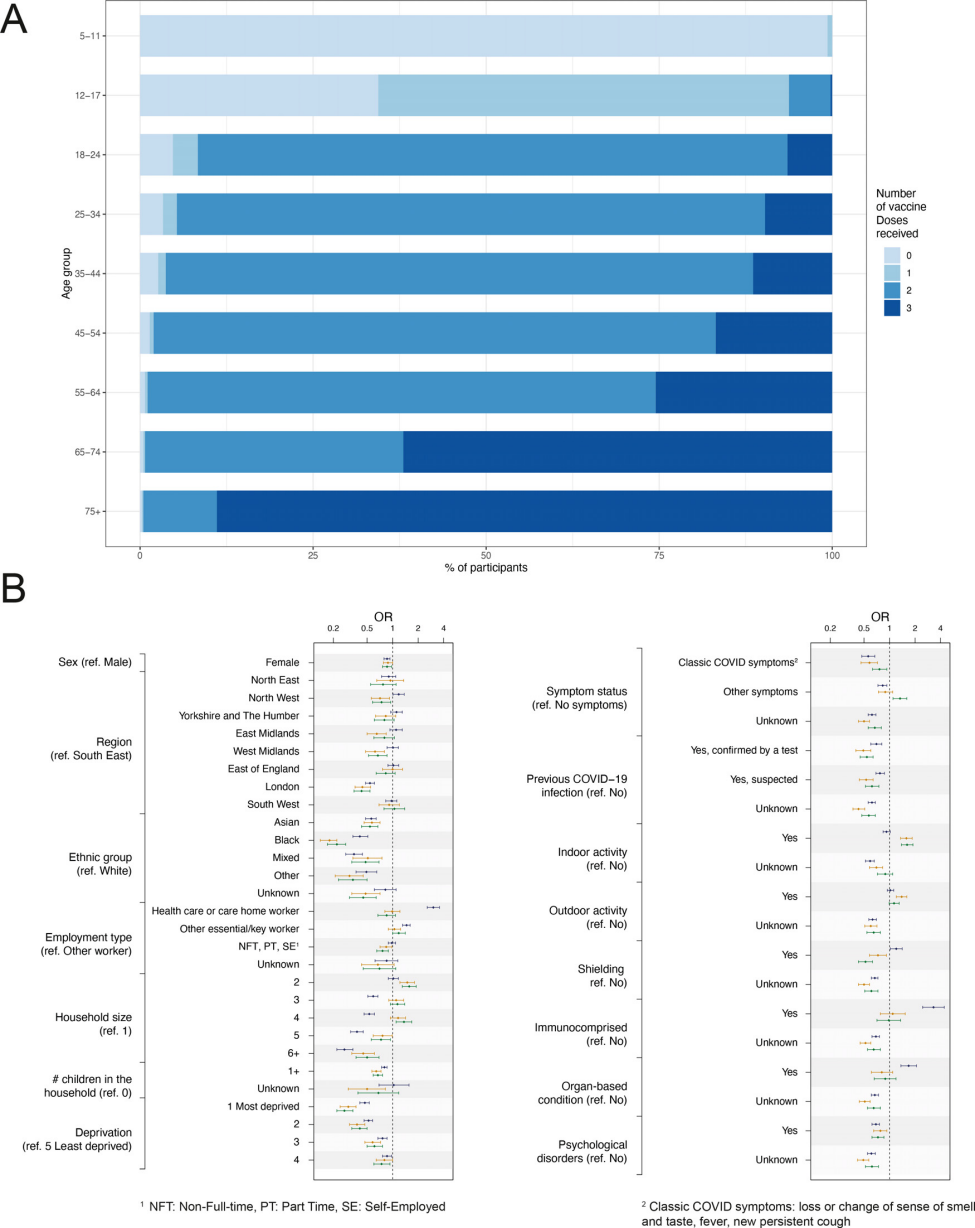
Vaccination uptake in England in November 2022. Proportion of unvaccinated (pale blue) participants and participants having received one, two, or three vaccine doses by age in round 15 **(A).** Results are based on linked vaccination data in consenting participants (children aged 12 years were combined with those aged 13 to 17 years, as they were eligible for vaccination). Comparison of the characteristics of the REACT-1 participants aged 18 to 64 years who received two vaccine doses to those unvaccinated in rounds 13–15 **(B)**. For each variable, we present the point estimate and 95% confidence interval of the Odds Ratio (OR) from the logistic model for vaccination status. The model is parameterised such that ORs greater than 1 indicate a greater probability of being vaccinated. Results are presented for round 13 (blue, 24 June to 12 July 2021), round 14 (orange, 9 to 27 September 2021), and round 15 (green, 19 October to 5 November 2021).

Across rounds 13 (predominantly Delta), 14 and 15 (all Delta), vaccination data were obtained (with consent) from linkage to NHS data in 259,955 participants. Of these, 132,333 aged 18 to 64 years had received two vaccine doses at least 14 days prior to swabbing, and 5,025 were unvaccinated across rounds 13, 14 and 15 (Table 1). The number of unvaccinated adults aged 18-64 years decreased from 2,951 (7.7%) in round 13 to 1,039 (2.0%) in round 14 and 1,035 (2.1%) in round 15, indicating high vaccine uptake at these ages (Figure 3-A). Using a logistic model for vaccination status (two doses vaccinated vs. unvaccinated), we identified key demographic and other differences between the unvaccinated and vaccinated individuals, especially during round 14 and 15 (Figure 3-B), such that their comparison in vaccine effectiveness analyses may result in biased and non-interpretable estimates for that age group. We instead examined prevalence of swab-positivity by round and vaccination status (Table 1). Unweighted prevalence of infection in unvaccinated adults decreased from 1.49% (1.09%, 2.00%) in round 13 to 1.16% (0.60%, 2.02%) in round 15, while unweighted prevalence of breakthrough infections in those who had received two doses of vaccine more than doubled between round 13 and round 15 from 0.41% (0.35%,0.48%) to 1.10% (1.01%, 1.20%), respectively.

**Table 1:** Unweighted prevalence of swab-positivity by vaccination status and round. Results are presented for rounds 13, 14 and 15 of REACT-1 using linked vaccine status data, for participants aged 18 to 64 years.

Round	Vaccination Status^1^	Test Positive	Total	Unweighted Prevalence (95% CI)
Round 13 (24 Jun - 12 Jul 2021)	Unvaccinated	44	2,951	1.49% (1.09%, 2.00%)
	2 doses All vaccines	144	34,997	0.41% (0.35%, 0.48%)
	2 doses AZ	109	25,043	0.44% (0.36%, 0.52%)
	2 doses Moderna	0	24	0.00% (0.00%, 14.25%)
	2 doses Pfizer	26	6,526	0.40% (0.26%, 0.58%)
	2 doses Unknown	9	3,404	0.26% (0.12%, 0.50%)
Round 14 (9-27 Sep 2021)	Unvaccinated	13	1,039	1.25% (0.67%, 2.13%)
	2 doses All vaccines	328	50,016	0.66% (0.59%, 0.73%)
	2 doses AZ	248	31,538	0.79% (0.69%, 0.89%)
	2 doses Moderna	4	1,201	0.33% (0.09%, 0.85%)
	2 doses Pfizer	56	12,944	0.43% (0.33%, 0.56%)
	2 doses Unknown	20	4,333	0.46% (0.28%, 0.71%)
Round 15 (19 Oct – 5 Nov 2021)	Unvaccinated	12	1,035	1.16% (0.60%, 2.02%)
	2 doses All vaccines	520	47,320	1.10% (1.01%, 1.20%)
	2 doses AZ	383	30,472	1.26% (1.13%, 1.39%)
	2 doses Moderna	15	1,392	1.08% (0.60%, 1.77%)
	2 doses Pfizer	106	13,410	0.79% (0.65%, 0.96%)
	2 doses Unknown	16	2,046	0.78% (0.45%, 1.27%)

1 Vaccination status for linked data was defined using time since last vaccination. Unvaccinated are those not having received any vaccine dose or one dose less than 14 days before swabbing; double dose vaccinated are those having received their second dose 14 days or more before swabbing.

Vaccination data indicate that 65% of the children aged 12 to 17 years had received one or two vaccine doses by round 15 (Figure 2-A), and the unvaccinated and vaccinated children aged 12 to 17 years were broadly comparable (Supplementary Figure 3), although immunosuppressed children and those suffering from psychological disorders or organ-based conditions, particularly in round 13, were found more likely to have been vaccinated. Using data from rounds 13, 14 and 15 and adjusting for age, sex, IMD<, region and ethnicity we estimated the adjusted vaccine effectiveness against infection for one or two doses of Pfizer-BioNTech (after 14 days) in children aged 12 to 17 years to be 58.1% (45.4%, 67.9%). Higher estimates were found in those reporting symptoms at 64.8% (51.8%,74.3%). Vaccine effectiveness estimates were similar when only considering children having received a single vaccine dose. Estimates for round 15 only were slightly higher with a fully adjusted vaccine effectiveness against infection of 64.0% (50.9%, 73.6%) and 67.7% (53.8%, 77.5%) in all, and symptomatic children, respectively (Table 2).

**Table 2:** Vaccine effectiveness against infection for children aged 12 to 17 years. Estimates are reported for (i) round 13 to round 15, and (ii) round 15 of REACT-1. Estimates are based on a logistic model of swab positivity in (i) children having received one or two vaccine doses and (ii) children having received a single vaccine dose compared to unvaccinated children. Results are adjusted for age, sex, Index of Multiple Deprivation (IMD), region, and ethnicity. For the model based on data from rounds 13 (24 June to 12 July 2021), 14 (9 to 27 September 2021) and 15 (19 October to 5 November 2021), estimates are further adjusted for round.

	Dataset		Test negatives	Test positives	Vaccine Effectiveness (VE)⁎⁎⁎ (95% CI)
Rounds 13-15	All children	Unvaccinated	11,360	380	-
		1 or 2 doses	3,160	74	58.13% (45.44%, 67.87%)
		1 dose	2,770	71	54.94% (40.98%, 65.60%)
	Children reporting any symptom*	Unvaccinated	11,447	293	-
		1 or 2 doses	3,183	51	64.81% (51.82%, 74.30%)
		1 dose	2,792	49	62.22% (47.95%, 72.57%)
	Children reporting any of the classic COVID-19 symptoms⁎⁎	Unvaccinated	11,498	242	-
		1 or 2 doses	3,189	45	61.22% (45.66%, 72.33%)
		1 dose	2,798	43	58.56% (41.52%, 70.64%)
Round 15	All children	Unvaccinated	2,796	207	-
		1 or 2 doses	1,965	55	63.97% (50.92%, 73.55%)
		1 dose	1,774	53	61.53% (47.33%, 71.90%)
	Children reporting any symptom*	Unvaccinated	2,839	164	-
		1 or 2 doses	1,980	40	67.73% (53.78%, 77.47%)
		1 dose	1,788	39	65.12% (49.83%, 75.75%)
	Children reporting any of the classic COVID-19 symptoms⁎⁎	Unvaccinated	2,874	129	-
		1 or 2 doses	1,985	35	63.95% (46.82%, 75.56%)
		1 dose	1,793	34	61.21% (42.48%, 73.84%)

⁎ Children reporting any of the 29 surveyed symptoms in the month prior to swabbing.

⁎⁎ Children reporting any of loss or change of sense of smell or taste, fever, new persistent cough in the month prior to swabbing.

⁎⁎⁎ VE is estimated by comparing vaccinated children testing positives and those testing negative. For analyses on symptomatic children, we compare. symptomatic test positives to test negatives and asymptomatic positives.

The proportion of REACT-1 round 15 participant having received a third vaccine dose increased with age (Figure 3-A) and ranged from 16.8% in those aged 45 to 54 years to 88.9% in those aged 75 years and over. Univariable logistic models for vaccination status (2 vaccine doses vs unvaccinated) in adult participants aged 18 years and over from rounds 13, 14, and 15 showed that participants (i) from Black, Asian, mixed ethnicity; (ii) living in large households including 5 or more persons, and (iii) living in most deprived areas were (independently) less likely to have received two vaccine doses (Figure 3-B). We found fewer differences in the characteristics of REACT-1 participants aged 18 years and over having received a third vaccine dose and those who received two doses only (Supplementary Figure 4). Females, participants from Asian, mixed and unknown ethnicity, participants from households with one or more children, participants living in London, and immunocompromised people were more likely to have received a third vaccine dose, along with those suffering from organ-based diseases. Conversely, participants reporting COVID-19 symptoms and those having already had a (confirmed or suspected) COVID-19 infection were less likely to have received a third dose. Similar results, although with slightly weaker effect size estimates, were found when restricting the population to those aged 50 years and over and including healthcare and homecare workers. In adults aged 18 years and over, the fully adjusted OR of swab-positivity associated with a third (booster) dose compared to two doses of vaccine was estimated at OR=0.36 (0.25, 0.53) for all vaccines combined (Table 3). Similar results were obtained for adults aged 50 years and over together with health care workers and care home workers under 50 years of age with a fully adjusted OR for swab positivity of 0.37 (0.25, 0.56).

**Table 3:** Effect of a third vaccine dose on the risk of swab positivity. Estimates are presented for participants of REACT-1 in round 15 (19 October to 5 November 2021) who had received at least two vaccine doses. Estimates are obtained comparing swab positivity in those having received two vaccine doses at least six months prior to swabbing and those having received three vaccine doses. Results are presented for adults aged 18 years and over in round 15 and for those either aged 50 years and over or healthcare or care home workers. In the latter group estimates are given for all vaccines combined and for AZ and Pfizer-BioNTech separately. Odds Ratios (ORs) are adjusted for age, sex, Index of Multiple Deprivation (IMD), region, and ethnicity.

Dataset	Vaccine Type		Test negatives	Test positives	OR (95% CI)
18 years and over	All Vaccines	2 Doses*	19,452	196	-
		3 Doses⁎⁎	8,300	32	0.36 (0.25, 0.53)
Healthcare or care home worker or 50 years and over	All Vaccines	2 Doses*	18,064	174	-
		3 Doses⁎⁎	8,039	31	0.37 (0.25, 0.56)
	AZ	2 Doses*	9,403	102	-
		3 Doses⁎⁎	1,942	10	0.47 (0.24, 0.90)
	Pfizer	2 Doses*	7,185	65	-
		3 Doses⁎⁎	5,647	19	0.33 (0.20, 0.57)

⁎ For comparability purposes, participants having received two vaccine doses were restricted to those eligible for a third dose: whose second dose was administered > 180 days prior to swabbing.

⁎⁎ We considered that the effect of the third dose was effective 14 days after vaccination. Participants with two doses are thus defined as those having only received two vaccine doses or three doses with the third one administered less than 14 days prior to swabbing; participants with three doses are those who received their third vaccine dose 14 days prior to swabbing.

## Discussion

The REACT-1 programme has been providing timely data on the spread of SARS-CoV-2 in the population of England since lockdown during the first wave of the epidemic in May 2020.^2^ Our analyses for round 15 cover the period from mid-October to early November 2021 spanning the autumn half-term break in schools in England. Compared with the previous round of data collection in September 2021,^12^ the prevalence of swab positivity in the population had risen markedly, especially among school-aged children, where we observed rates averaging around 5% over the period. Although prevalence was somewhat lower in older people (65 years and over) it had approximately doubled between rounds, and unlike in younger people, did not appear to be falling during the period of the current study, despite high levels of vaccination in this group.

Our study confirmed that Delta variant and its sub-lineages was the dominant strain circulating in England to early November 2021, as has also been reported by the UK Health Security Agency (zS) (UKHSA) based on the national routine testing programme.^24^ Our data suggest that against the backdrop of Delta and the removal of all restrictions in England, viral transmission increased from September to mid-October 2021, with the subsequent fall in swab positivity being driven by the younger ages, possibly related, at least in part, to the half-term break. The increase in prevalence in older people suggests that transmission can be sustained among the vaccinated as well as the unvaccinated population. A recent study of transmission in the home related to Delta virus suggested only a modest reduction in risk of infection among double-vaccinated compared to unvaccinated individuals, with secondary attack rates of 25% (95% CI 18%, 33%) and 38% (24%, 53%), respectively.^6^ In keeping with these results, we found that people living in larger households and those with children in the household had higher prevalence of swab-positivity than single-person households and those without children.^12^

In previous rounds we estimated vaccine effectiveness among adults at ages 18 to 64 years,^12^ but not older adults, because of concern – given so few were unvaccinated – that the unvaccinated group might be non-representative of the wider population. Estimating vaccine effectiveness from observational data is well known to run the risk of bias arising from the non-comparability of the vaccinated and unvaccinated groups in key aspects other than vaccination. For example, “confounding by indication” can arise if the presence of one or more underlying conditions affects an individual's decision to get vaccinated, while a “healthy vaccine bias” may occur where individuals who are healthier are more likely to accede to the vaccination programme.^12^ These differential effects can bias estimates of effectiveness in either direction even with statistical adjustments for measured covariates, in an attempt to correct for non-vaccination-related differences between the vaccinated and unvaccinated groups.^25^ New approaches are being developed to try to tease out such effects.^26^

Our concern about estimating vaccine effectiveness among adults at ages 65 years and over due to non-comparability between the vaccinated and (small) unvaccinated group also became a key consideration here at ages 18 to 64 years. First, reflecting the success of the vaccine programme, only a small proportion of this age group remained unvaccinated in the present round, resulting in wide confidence intervals and imprecise estimates of prevalence in the unvaccinated (comparator) group, especially in the latter rounds. Second, there was possible waning in immunity following vaccination that may have led to increasing risk of breakthrough infections among the double-vaccinated group. Finally, there was evidence that the unvaccinated and double-vaccinated groups differed in important ways according to a variety of parameters, suggesting that the unvaccinated and vaccinated groups were poorly matched.

In contrast, estimates of the effects of the third vaccine dose on swab positivity, compared to two doses, were not affected by these issues in the same way. Since those receiving a third dose of vaccine were already double-vaccinated, they were as a group likely to be much more closely matched to the double-vaccinated group than comparisons of vaccinated and unvaccinated individuals. This was even more so given that the roll-out and scale-up of the booster vaccine programme during the round meant that many people who wanted to get a third vaccine dose were still waiting their turn. We estimated that following a third vaccine dose, the odds of swab-positivity were on average around one third of the odds of double-vaccinated individuals, indicating an effective immune response against Delta variant from the third dose. However, our estimate is less strong than that reported for symptomatic individuals in both Israel^10^ and the UK^27^ both of which depended on people presenting with symptoms to the national testing programmes. Our results use vaccination data linked to personal NHS records in consenting participants (>85%) and are community-based, include non-symptomatic individuals and are not contingent on test-seeking behaviours. As such our more prudent estimates may better capture the heterogeneity in the uptake and effectiveness of the vaccination programme in the community, and thus may offer more realistic and generalisable view of the situation in England and other countries in the UK and Europe.

Our data enabled us to investigate the effect of vaccination in children aged 12 to 17 years in England. Our results show the benefits of single doses delivered to children aged 12 years and above in terms of reducing the risk of swab positivity and by extension onward transmission of infection. Preventing infections is important because, as already noted, those with breakthrough infections post-vaccination do transmit to others, including within households at a rate comparable to those who are infected but have not been vaccinated.^6^ In addition, high rates of infections in schools are disruptive to learning and education.

Crucially, the vaccination programme has extensively reduced the risks of COVID-19 related hospitalisations and deaths.^28^ In January 2021 when the average swab-positivity prevalence in REACT-1 was similar to that recorded in the current round, hospitalisations in England were running at around 3,500 per day compared with around 850 per day at the beginning of November 2021.^29^ Looking further at our own data, we can observe a weakening of the association between swab-positivity in REACT-1 and COVID-19 related hospitalisations and deaths arising on average 19 and 25 days later. Nonetheless the booster programme in the UK needs to reach the vast majority of the more vulnerable population to prevent further pressure on health services from waning immunity.

Our study has limitations. Although the response rate in REACT-1 has declined steadily from 30.5% in round 1 to its current level of 11.7% in round 15, we use rim weighting to obtain prevalence estimates that are representative of the population of England as a whole. While we obtain vaccination data reported by participants, here we relied on data on vaccination as recorded by the NHS for the >85% who gave permission to link to their NHS records. This has the advantage of providing accurate information on the date of vaccination and type of vaccine used, both important to avoid errors and biases in estimates of prevalence and vaccine effectiveness, with no dependency on participant recall. However, to the extent that those who consent and do not consent to data linkage may differ (although the large proportion of people do consent to linkage), and those who are vaccinated and unvaccinated may have differing propensities to take part, it is possible that undetected systematic errors may be introduced. Finally, we changed the method by which swabs were sent to the laboratory for RT-PCR in round 15, relying on the priority postal service for participants to return their swab (transported in saline solution). In the previous round we tested in a 1:1 randomised fashion either courier pick-up (no cold chain) or priority postal service and found no discernible difference between the two in either positivity rate or Ct values.^12^ Prior to that, dry swabs were sent by courier to the laboratory on a cold chain. Sampling of participants was performed with replacement in REACT-1 resulting in a small number of participants being included in more than one round: 46 (0.05%) and 67 (0.07%) participants included in round 15 were also included in rounds 13 and 14, respectively. Data from rounds 13, 14 and 15 should therefore not be considered completely independent. However, the limited number of overlapping participants, the fact that participants sampled in each round (including those sampled more than once) were selected to be representative of the resident population of England as a whole, and our use of rim weighting to correct for potential sampling bias, should protect our results against meaningful bias or between-round correlation when comparing and/or pooling data from round 14, 15, and 16.

In conclusion, swab-positivity was high at the start of round 15 in mid-October 2021, reaching a maximum around 20 to 21 October 2021, and then falling through late October with an uncertain trend in the last few days of data collection in early November 2021. School-aged children were the most likely to test positive, while at the same time children who had received a single dose of the Pfizer-BioNTech vaccine at least 14 days prior to swabbing had around 50% reduced risk of infection compared to unvaccinated children of the same age (12 to 17 years). Likewise, booster (third) vaccine doses were found to protect adults from infection, compared to their counterparts who had received only two doses. The relatively small proportion of adults who remain unvaccinated (a clear positive from a public health perspective) limited our ability to reliably estimate vaccine effectiveness by comparing those who had received two doses of the vaccine to those remaining unvaccinated. Thus, we cannot usefully add to our previous report^12^ that showed the protection offered by two vaccine doses waned over time. Expanded availability and rapid roll-out of booster doses, second doses for teenagers aged 16 to 17 years and single doses for children aged 12 to 15 years in England, with possible extension to younger children, should help reduce transmission from Delta and other variants during the winter period when healthcare demands typically rise. At the time of data collection, vaccinations were not approved for children aged under 12 years in England. However, based on results from a clinical trial in that age group,^30^ the Joint Committee on Vaccination and Immunisation recommended vaccination of children aged 5 to 11 years in England in late December 2022.^31^ Overall, data from the different rounds of REACT-1 provide unbiased estimates of the prevalence of SARS-CoV-2 swab-positivity in the resident population at different stages of the epidemic and, combining data across rounds, enable the assessment of the dynamics of growth/decay of the epidemic. Linkage to NHS vaccination data allowed us to accurately estimate vaccine effectiveness against infection. While the present report focussed on the epidemiological situation in October 2021, when Delta predominated, extending these analyses to subsequent rounds of the REACT-1 study will provide population-based and policy-relevant estimates of the prevalence of the infection for different age groups, and for different viral strains.

### Contributors

PE and CAD are corresponding authors. PE, MC-H and CAD conceived the study and the analytical plan. MC-H, OE, BB, HWang, DH, JJ and CEW performed the statistical analyses. HWang, OE, DH, BB, and MW curated the data. JE, CA, PJD, DA, WB, GT, GC, HWard, AD provided study oversight and results interpretation. AJP generated the sequencing data. AD and PE obtained funding. All authors revised the manuscript for important intellectual content and approved the submission of the manuscript.

## Funding

Department of Health and Social Care in England

### Data sharing statement

Access to REACT-1 individual-level data is restricted to protect participants’ anonymity.

Summary statistics, descriptive tables, and code from the current REACT-1 study are available at https://github.com/mrc-ide/reactidd (doi 10.5281/zenodo.6242826). REACT-1 study materials are available for each round at https://www.imperial.ac.uk/medicine/research-and-impact/groups/react-study/react-1-study-materials/ Sequence read data are available without restriction from the European Nucleotide Archive at https://www.ebi.ac.uk/ena/browser/view/PRJEB37886, and consensus genome sequences are available from the Global initiative on sharing all influenza data (GISAID).

## Declaration of interests

Prof. Elliott is the director of the MRC Centre of Environment and Health (MR/L01341X/1 and MC/S019669/1) and the NIHR Health Protection Research Unit in Chemical and Radiation Threats and Hazards. Prof M Chadeau-Hyam holds shares in the O-SMOSE company. Consulting activities conducted by the company are independent of the present work. All other authors have no conflict of interest to disclose.

## Appendix Supplementary materials

Image, application 1
