# Supplementary material for: Breakthrough SARS-CoV-2 infections in double and triple vaccinated adults and single dose vaccine effectiveness among children in Autumn 2021 in England: REACT-1 study
Source: eClinicalMedicine. 2022 May 6;48:101419. doi: 10.1016/j.eclinm.2022.101419 (PMC9076030; doi:10.1016/j.eclinm.2022.101419)
Supplement: Supplementary file 1 [file mmc1.pdf]

Supplementary Information:

Breakthrough SARS-CoV-2 infections in vaccinated adults, vaccine effectiveness against infection for booster doses in adults and single dose in children in Autumn 2021 in England: REACT-1 study

Short title: SARS-CoV-2 breakthrough infection and vaccination during Autumn 2021 in England

Supplementary Tables and Figures

Supplementary Table 1. Unweighted and weighted prevalence of swab-positivity from REACT-1 across rounds 1 to 15.

Round	Tested swabs	Positive swabs	Unweighted prevalence (95% CI)	Weighted prevalence (95% CI)	First sample	Last sample
1	120,620	159	0.13% (0.11%, 0.15%)	0.16% (0.13%, 0.19%)	01/05/20	01/06/20
2	159,199	123	0.08% (0.07%, 0.09%)	0.09% (0.07%, 0.11%)	19/06/20	07/07/20
3	162,821	54	0.03% (0.03%, 0.04%)	0.04% (0.03%, 0.05%)	24/07/20	11/08/20
4	154,325	137	0.09% (0.08%, 0.11%)	0.13% (0.01%, 0.15%)	20/08/20	08/09/20
5	174,949	824	0.47% (0.44%, 0.50%)	0.60% (0.55%, 0.71%)	18/09/20	05/10/20
6	160,175	1,732	1.08% (1.03%, 1.13%)	1.30% (1.21%, 1.39%)	16/10/20	02/11/20
7	168,181	1,299	0.77% (0.73%, 0.82%)	0.94% (0.87%, 1.01%)	13/11/20	03/12/20
8	167,642	2,282	1.36% (1.31%, 1.42%)	1.57% (1.49%, 1.66%)	06/01/21	22/01/21
9	165,456	689	0.42% (0.39%, 0.45%)	0.49% (0.44%, 0.55%)	04/02/21	23/02/21
10	140,844	227	0.16% (0.14%, 0.18%)	0.20% (0.17%, 0.23%)	11/03/21	30/03/21
11	127,408	115	0.09% (0.07%, 0.11%)	0.10% (0.08%, 0.13%)	15/04/21	03/05/21
12*	108,911	135	0.12% (0.10%, 0.15%)	0.15% (0.12%, 0.18%)	20/05/21	07/06/21
13	98,233	527	0.54% (0.49%, 0.58%)	0.63% (0.57%, 0.69%)	24/06/21	12/07/21
14**	100,527	764	0.76% (0.71%, 0.82%)	0.83% (0.76%, 0.89%)	09/09/21	27/09/21
15***	100,112	1,399	1.40% (1.33%, 1.47%)	1.57% (1.48%, 1.66%)	19/10/21	05/11/21

* Sampling strategy changed for round 12 and subsequent rounds. Therefore unweighted prevalence is not directly comparable with previous rounds
** Including N=509 samples from 28-30 September. Sample handling changed in round 14. Prevalence is not directly comparable with previous rounds
*** Including N=93 samples (all negatives) from 6-8 November, and N=86 samples with no collection/arrival dates

Supplementary Table 2. Unweighted and weighted prevalence of swab-positivity in round (9 to 27 September 2021) and round 15 (19 October to 5 November 2021) by sex, age, region, employment type, and ethnic group.

		Round 14				Round 15			
Variable		Positive	Total	Unweighted Prevalence	Weighted Prevalence	Positive	Total	Unweighted Prevalence	Weighted Prevalence
Sex	Male	336	44,549	0.75% (0.68%, 0.84%)	0.82% (0.73%, 0.92%)	679	44,887	1.51% (1.40%, 1.63%)	1.66% (1.53%, 1.80%)
	Female	428	55,976	0.76% (0.69%, 0.84%)	0.83% (0.75%, 0.93%)	720	55,223	1.30% (1.21%, 1.40%)	1.48% (1.36%, 1.60%)
	Unknown	0	2			0	2		
Age	05-12	155	6,458	2.40% (2.04%, 2.80%)	2.32% (1.96%, 2.73%)	277	5,702	4.86% (4.31%, 5.45%)	4.95% (4.39%, 5.58%)
	13-17	118	4,927	2.40% (1.99%, 2.86%)	2.55% (2.11%, 3.08%)	283	5,394	5.25% (4.67%, 5.88%)	5.21% (4.61%, 5.87%)
	18-24	9	2,452	0.37% (0.17%, 0.70%)	0.46% (0.23%, 0.90%)	16	3,088	0.52% (0.30%, 0.84%)	0.56% (0.33%, 0.95%)
	25-34	28	7,374	0.38% (0.25%, 0.55%)	0.36% (0.24%, 0.53%)	60	7,781	0.77% (0.59%, 0.99%)	0.81% (0.62%, 1.07%)
	35-44	100	12,118	0.83% (0.67%, 1.00%)	0.79% (0.64%, 0.97%)	160	11,933	1.34% (1.14%, 1.56%)	1.29% (1.09%, 1.51%)
	45-54	130	16,855	0.77% (0.64%, 0.92%)	0.78% (0.65%, 0.93%)	209	16,459	1.27% (1.10%, 1.45%)	1.32% (1.14%, 1.52%)
	55-64	113	20,856	0.54% (0.45%, 0.65%)	0.55% (0.45%, 0.67%)	177	20,924	0.85% (0.73%, 0.98%)	0.87% (0.74%, 1.01%)
	65-74	80	19,313	0.41% (0.33%, 0.52%)	0.42% (0.34%, 0.53%)	157	19,687	0.80% (0.68%, 0.93%)	0.84% (0.72%, 0.99%)
	75+	31	10,174	0.30% (0.21%, 0.43%)	0.29% (0.20%, 0.42%)	60	9,144	0.66% (0.50%, 0.84%)	0.63% (0.48%, 0.82%)
Region	South East	92	17,388	0.53% (0.43%, 0.65%)	0.57% (0.45%, 0.72%)	261	17,637	1.48% (1.31%, 1.67%)	1.62% (1.41%, 1.84%)
	North East	39	4,551	0.86% (0.61%, 1.17%)	0.84% (0.60%, 1.18%)	64	4,479	1.43% (1.10%, 1.82%)	1.79% (1.36%, 2.34%)
	North West	121	12,117	1.00% (0.83%, 1.19%)	0.99% (0.81%, 1.21%)	179	12,079	1.48% (1.27%, 1.71%)	1.65% (1.40%, 1.95%)
	Yorkshire and The Humber	105	9,887	1.06% (0.87%, 1.28%)	1.25% (1.00%, 1.57%)	136	9,720	1.40% (1.18%, 1.65%)	1.43% (1.18%, 1.73%)
	East Midlands	87	8,830	0.99% (0.79%, 1.21%)	1.15% (0.92%, 1.44%)	110	8,647	1.27% (1.05%, 1.53%)	1.47% (1.20%, 1.80%)
	West Midlands	88	10,249	0.86% (0.69%, 1.06%)	1.01% (0.80%, 1.27%)	135	9,943	1.36% (1.14%, 1.60%)	1.59% (1.32%, 1.91%)
	East of England	87	11,756	0.74% (0.59%, 0.91%)	0.73% (0.59%, 0.92%)	158	11,560	1.37% (1.16%, 1.60%)	1.57% (1.32%, 1.86%)
	London	91	14,885	0.61% (0.49%, 0.75%)	0.62% (0.50%, 0.79%)	160	14,847	1.08% (0.92%, 1.26%)	1.23% (1.03%, 1.47%)
	South West	54	10,864	0.50% (0.37%, 0.65%)	0.59% (0.43%, 0.80%)	196	11,200	1.75% (1.52%, 2.01%)	1.97% (1.69%, 2.29%)
Employment type	Health care or care home worker	59	7,963	0.74% (0.56%, 0.95%)	0.80% (0.60%, 1.06%)	92	7,649	1.20% (0.97%, 1.47%)	1.53% (1.23%, 1.91%)
	Other essential/key worker	159	14,627	1.09% (0.93%, 1.27%)	1.07% (0.90%, 1.28%)	237	14,065	1.68% (1.48%, 1.91%)	1.87% (1.63%, 2.15%)
	Other worker	263	38,496	0.68% (0.60%, 0.77%)	0.71% (0.62%, 0.82%)	476	38,433	1.24% (1.13%, 1.35%)	1.38% (1.25%, 1.53%)
	Not full-time, part-time, or self-employed	244	37,312	0.65% (0.57%, 0.74%)	0.77% (0.67%, 0.89%)	518	37,892	1.37% (1.25%, 1.49%)	1.53% (1.39%, 1.68%)
	Unknown	39	2,129	1.83% (1.31%, 2.50%)	1.88% (1.34%, 2.64%)	76	2,073	3.67% (2.90%, 4.57%)	3.50% (2.76%, 4.44%)
Ethnic group	White	635	87,942	0.72% (0.67%, 0.78%)	0.78% (0.72%, 0.85%)	1,202	87,741	1.37% (1.29%, 1.45%)	1.53% (1.44%, 1.63%)
	Asian	58	5,550	1.04% (0.79%, 1.35%)	1.04% (0.77%, 1.41%)	91	5,243	1.74% (1.40%, 2.13%)	2.00% (1.58%, 2.53%)
	Black	22	1,947	1.13% (0.71%, 1.71%)	1.41% (0.91%, 2.19%)	34	1,919	1.77% (1.23%, 2.47%)	1.66% (1.16%, 2.37%)
	Mixed	18	1,754	1.03% (0.61%, 1.62%)	1.01% (0.62%, 1.63%)	38	1,781	2.13% (1.51%, 2.92%)	2.24% (1.59%, 3.14%)
	Other	12	1,015	1.18% (0.61%, 2.06%)	1.01% (0.56%, 1.82%)	12	1,074	1.12% (0.58%, 1.94%)	1.22% (0.67%, 2.22%)
	Unknown	19	2,319	0.82% (0.49%, 1.28%)	1.09% (0.67%, 1.75%)	22	2,354	0.93% (0.59%, 1.41%)	1.09% (0.69%, 1.71%)

Supplementary Table 3. Growth rates, reproduction numbers and doubling/halving times from exponential model fits on data from round 15 (19 October to 5 November 2021 only (top table) and round 14 (9 to 27 September 2021) and round 15 (bottom table).

Rounds			Growth rate	R	Probability R>1	Doubling (+) / Halving (-) time
15	All positives		-0.040 (-0.053 , -0.028)	0.76 (0.70 , 0.83)	<0.01	-17.2 (-13.2 , -24.7)
	Subset	Non-symptomatics	-0.057 (-0.081 , -0.033)	0.68 (0.56 , 0.80)	<0.01	-12.3 (-8.6 -21.1)
		Symptomatics	-0.063 (-0.079 , -0.047)	0.64 (0.56 , 0.72)	<0.01	-10.9 (-8.7 , -14.6)
		Positive for both E and N genes	-0.045 (-0.058 , -0.032)	0.74 (0.67 , 0.81)	<0.01	-15.4 (-12.0 , -21.4)
		Positive for both E and N genes or positive only for N gene with CT 35 or less	-0.046 (-0.058 , -0.033)	0.73 (0.67 , 0.80)	<0.01	-15.2 (-12.0 , -21.0)
	Age	Aged 17 and under	-0.056 (-0.074 , -0.039)	0.68 (0.59 , 0.77)	<0.01	-12.3 (-9.4 , -17.7)
		Aged 18 to 54	-0.033 (-0.054 , -0.011)	0.80 (0.69 , 0.93)	<0.01	-21.0 (-12.8 , *)
		Aged 55 and over	0.003 (-0.026 , 0.033)	1.02 (0.84 , 1.22)	0.59	* (-26.9 , 21.2)
	Region	East Midlands	-0.083 (-0.128 , -0.039)	0.55 (0.36 , 0.77)	<0.01	-8.3 (-5.4 , -17.7)
		West Midlands	-0.038 (-0.076 , 0.000)	0.77 (0.58 , 1.00)	0.02	-18.2 (-9.1 , *)
		East of England	-0.032 (-0.068 , 0.004)	0.81 (0.62 , 1.03)	0.04	-21.7 (-10.2 , *)
		London	-0.022 (-0.057 , 0.012)	0.87 (0.67 , 1.08)	0.11	-32.1 (-12.2 , *)
		North West	-0.035 (-0.070 , -0.001)	0.79 (0.61 , 0.99)	0.02	-19.7 (-10.0 , *)
		North East	-0.029 (-0.086 , 0.027)	0.82 (0.53 , 1.18)	0.15	-23.5 (-8.0 , 25.7)
		South East	-0.050 (-0.079 , -0.021)	0.71 (0.57 , 0.87)	<0.01	-13.8 (-8.8 , -32.8)
		South West	-0.049 (-0.083 , -0.015)	0.72 (0.55 , 0.91)	<0.01	-14.1 (-8.3 , -47.1)
		Yorkshire and The Humber	-0.010 (-0.051 , 0.032)	0.94 (0.70 , 1.22)	0.33	* (-13.5 , 21.4)
14-15	All positives		0.014 (0.012 , 0.017)	1.09 (1.08 , 1.11)	>0.99	48.0 (* , 41.9)
	Subset	Non-symptomatics	0.016 (0.011 , 0.020)	1.10 (1.07 , 1.13)	>0.99	44.3 (* 34.9)
		Symptomatics	0.013 (0.011 , 0.016)	1.09 (1.07 , 1.11)	>0.99	* (* , 42.8)
		Positive for both E and N genes	0.014 (0.012 , 0.016)	1.09 (1.08 , 1.10)	>0.99	* (* , 43.3)
		Positive for both E and N genes or positive only for N gene with CT 35 or less	0.014 (0.012 , 0.016)	1.09 (1.08 , 1.10)	>0.99	* (* , 43.3)
	Age	Aged 17 and under	0.017 (0.014 , 0.020)	1.11 (1.09 , 1.13)	>0.99	41.7 (* , 35.1)
		Aged 18 to 54	0.011 (0.008 , 0.015)	1.07 (1.05 , 1.10)	>0.99	* (* , 46.4)
		Aged 55 and over	0.015 (0.010 , 0.019)	1.09 (1.06 , 1.13)	>0.99	47.7 (* , 35.7)
	Region	East Midlands	0.005 (-0.002 , 0.012)	1.03 (0.99 , 1.08)	0.93	* (* , *)
		West Midlands	0.010 (0.003 , 0.016)	1.06 (1.02 , 1.10)	>0.99	* (* , 43.5)
		East of England	0.018 (0.011 , 0.024)	1.12 (1.07 , 1.16)	>0.99	38.8 (* , 28.3)
		London	0.017 (0.011 , 0.024)	1.11 (1.07 , 1.16)	>0.99	40.3 (* , 29.3)
		North West	0.012 (0.006 , 0.017)	1.08 (1.04 , 1.11)	>0.99	* (* , 39.9)
		North East	0.016 (0.007 , 0.026)	1.11 (1.04 , 1.17)	>0.99	42.5 (* , 26.6)
		South East	0.023 (0.017 , 0.028)	1.15 (1.11 , 1.19)	>0.99	30.7 (41.0 , 24.4)
		South West	0.027 (0.020 , 0.034)	1.18 (1.13 , 1.23)	>0.99	25.4 (34.0 , 20.1)
		Yorkshire and The Humber	0.003 (-0.004 , 0.009)	1.02 (0.98 , 1.06)	0.79	* (* , *)

* Doubling/Halving time had an estimated magnitude greater than 50 days and so represented approximately constant prevalence

Supplementary Table 4. Proportion of each Delta sub-lineage detected in 841 positive samples from round 15 (19 October to 5 November 2021).

Sub-lineage	N (841)	Proportion	
B.1.617.2 (Delta/Sub-lineage not known)	108	0.128	(0.107 , 0.153)
AY.109	1	0.001	(0.000 , 0.007)
AY.110	1	0.001	(0.000 , 0.007)
AY.111	3	0.004	(0.001 , 0.010)
AY.25	1	0.001	(0.000 , 0.007)
AY.33	1	0.001	(0.000 , 0.007)
AY.34	6	0.007	(0.003 , 0.015)
AY.39	7	0.008	(0.004 , 0.017)
AY.4	484	0.576	(0.542 , 0.608)
AY.4.2	99	0.118	(0.098 , 0.141)
AY.4.5	4	0.005	(0.002 , 0.012)
AY.42	5	0.006	(0.003 , 0.014)
AY.43	40	0.048	(0.035 , 0.064)
AY.44	13	0.015	(0.009 , 0.026)
AY.46.2	3	0.004	(0.001 , 0.010)
AY.46.5	6	0.007	(0.003 , 0.015)
AY.47	2	0.002	(0.001 , 0.009)
AY.5	21	0.025	(0.016 , 0.038)
AY.5.4	1	0.001	(0.000 , 0.007)
AY.57	1	0.001	(0.000 , 0.007)
AY.6	13	0.015	(0.009 , 0.026)
AY.60	1	0.001	(0.000 , 0.007)
AY.7.2	1	0.001	(0.000 , 0.007)
AY.79	1	0.001	(0.000 , 0.007)
AY.80	1	0.001	(0.000 , 0.007)
AY.88	1	0.001	(0.000 , 0.007)
AY.9	2	0.002	(0.001 , 0.009)
AY.9.1	2	0.002	(0.001 , 0.009)
AY.9.2	1	0.001	(0.000 , 0.007)
AY.90	1	0.001	(0.000 , 0.007)
AY.91	1	0.001	(0.000 , 0.007)
AY.98	9	0.011	(0.006 , 0.020)

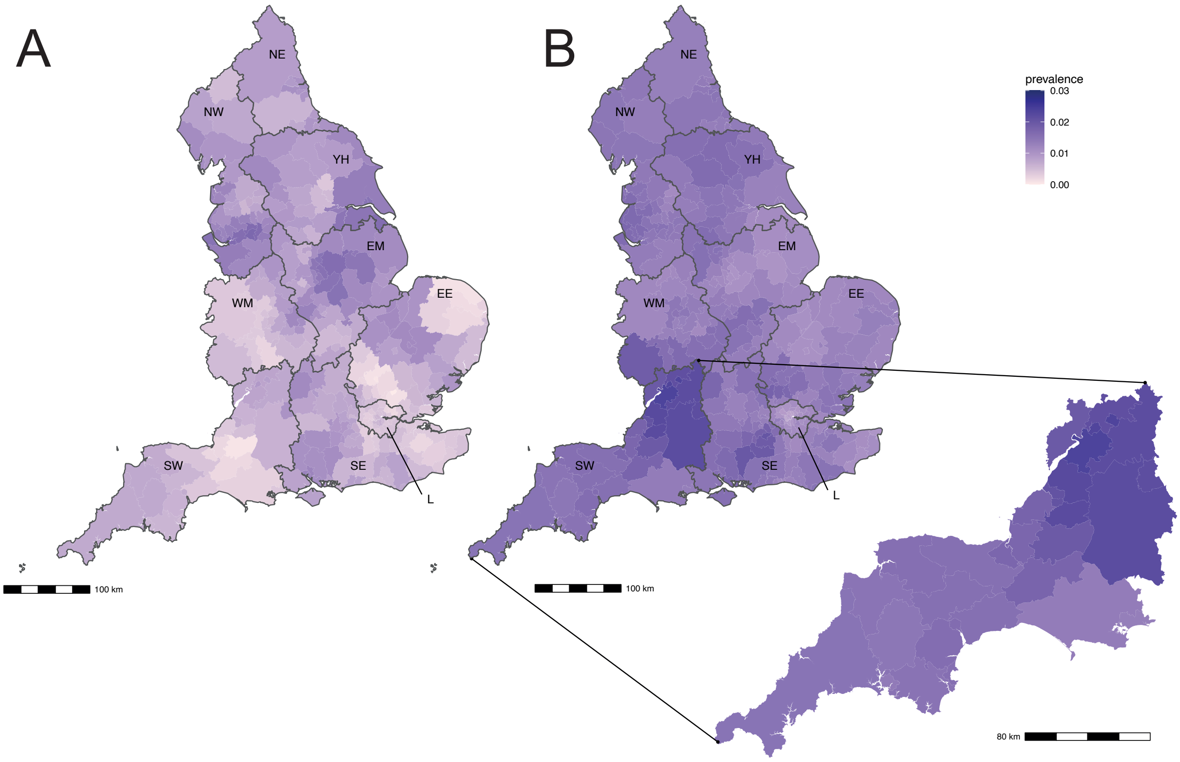

Supplementary Figure 1. Neighbourhood smoothed average prevalence by lower tier local authority area for round 14 (9 to 27 September 2021) **(A)** and round 15 (19 October to 5 November 2021) **(B)**. Neighbourhood prevalence calculated from nearest neighbours (the median number of neighbours within 30 km in the study). Average neighbourhood prevalence displayed for individual lower-tier local authorities. Regions: NE = North East, NW = North West, YH = Yorkshire and The Humber, EM = East Midlands, WM = West Midlands, EE = East of England, L = London, SE = South East, SW = South West. The 10 LTLAs with highest smoothed prevalence were all in South West: Stroud, Cheltenham, Gloucester, South Gloucestershire, Bath and North East Somerset, Wiltshire, Cotswold, Swindon, Tewkesbury, and City of Bristol.

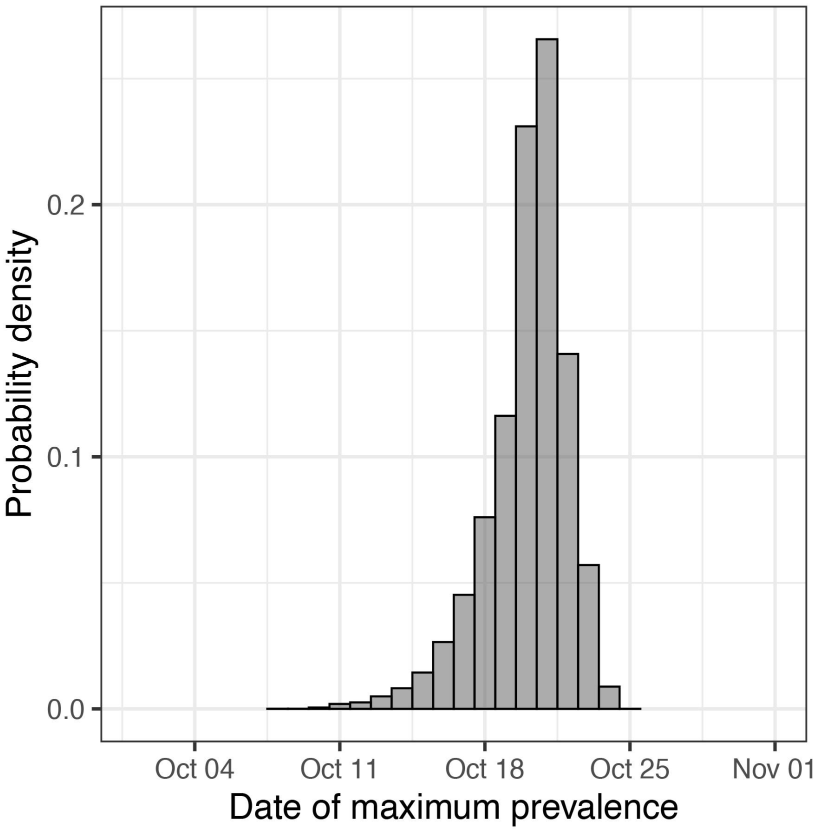

Supplementary Figure 2. Posterior probability density for the estimated date at which weighted prevalence, as estimated from the P-spline model, was at a maximum during the period of round 14 to round 15.

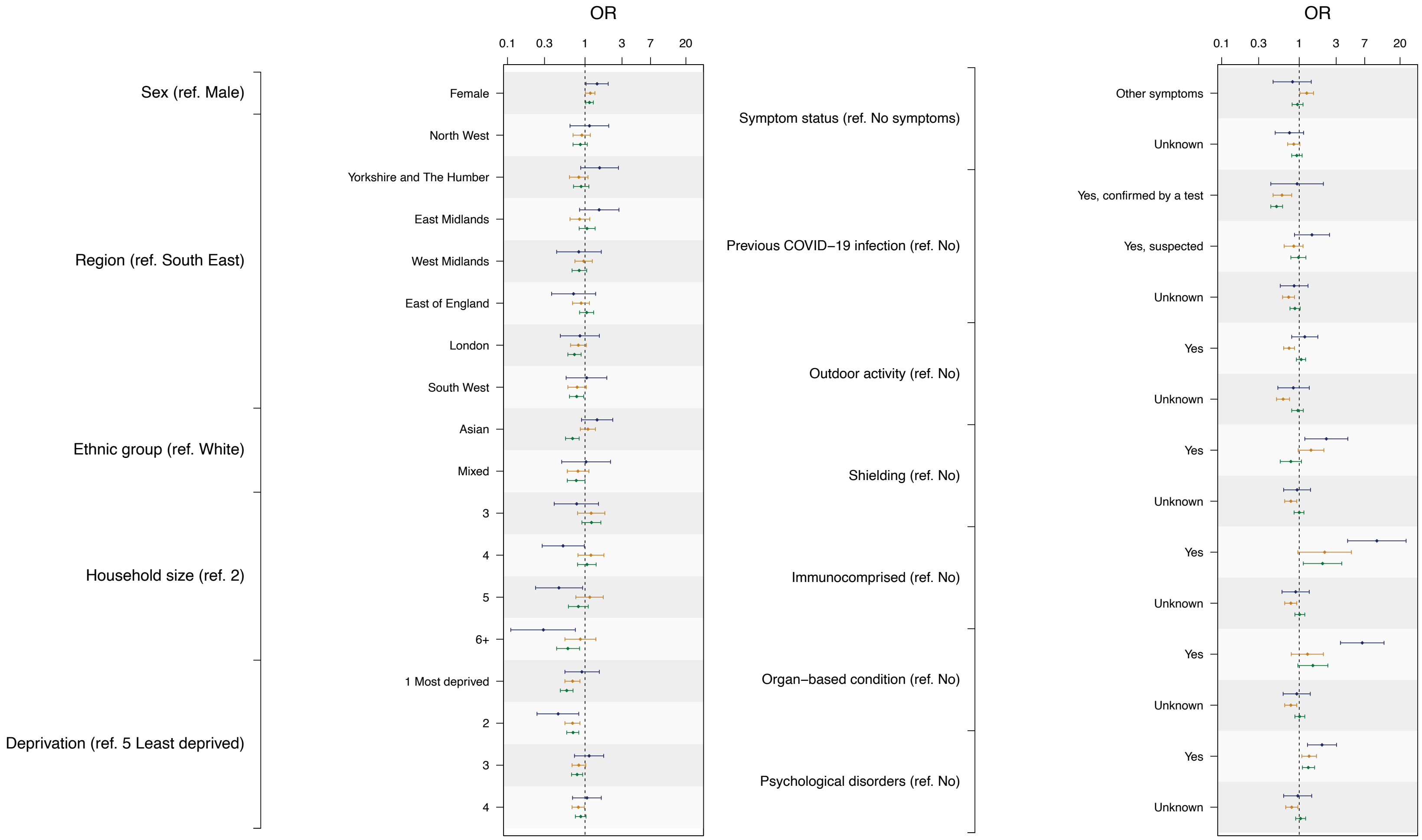

Supplementary Figure 3. Comparison of the characteristics of the REACT-1 single-or-double vaccinated to unvaccinated children aged 12 to 17 in rounds 13-15. For each variable, we present the point estimate and 95% confidence interval of the Odds Ratio (OR) from the logistic model for vaccination status. The model is parameterised such that ORs greater than 1 indicate a greater probability of being vaccinated. Results are presented for round 13 (blue, 24 June to 12 July 2021), round 14 (orange, 9 to 27 September 2021), and round 15 (green, 19 October to 5 November 2021).

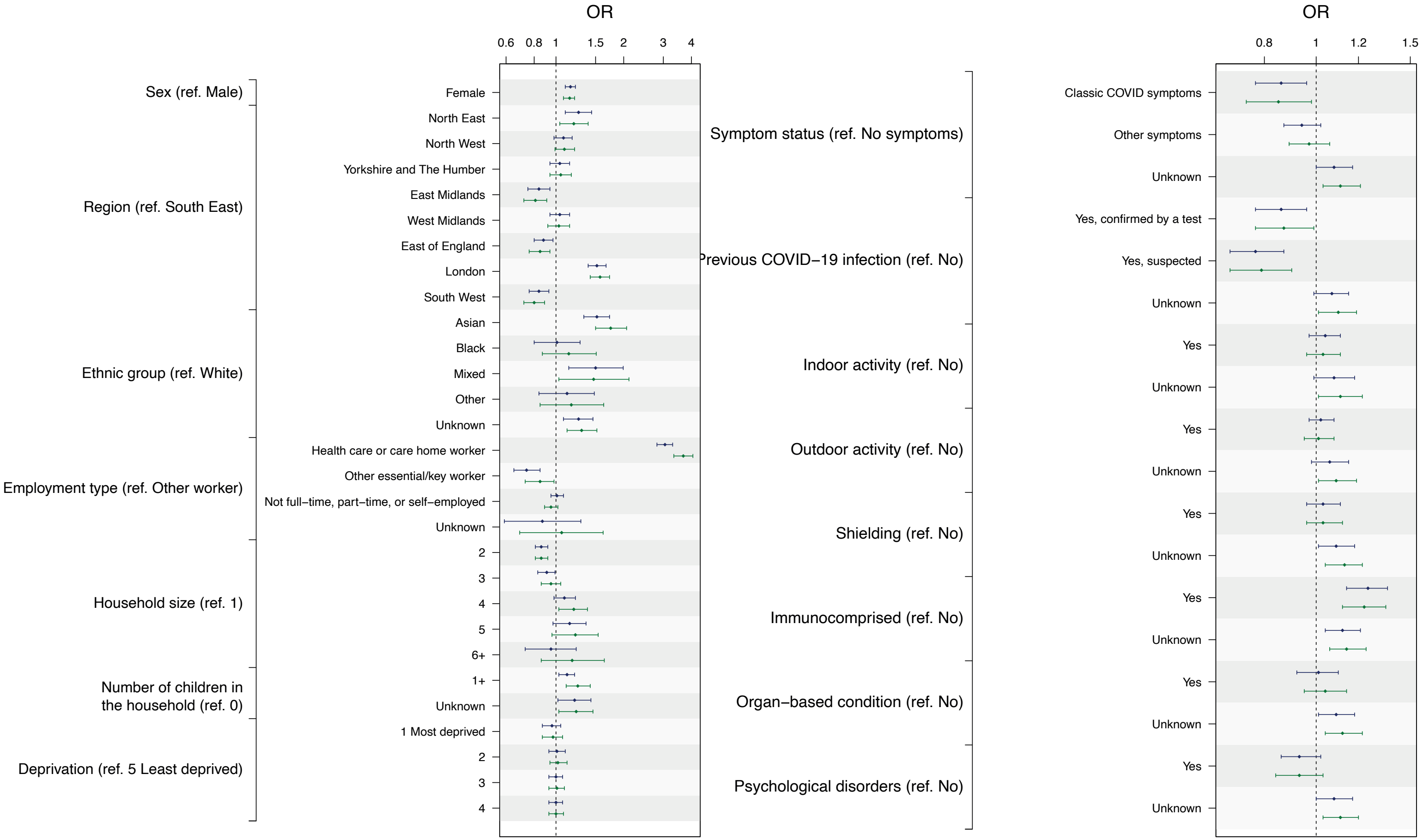

Supplementary Figure 4. Comparison of the characteristics of the REACT-1 participants who received a third vaccine dose to those having received two vaccine doses. For comparability purposes, participants with two vaccine doses are restricted to those who were eligible for a third vaccine dose (i.e. who received their second vaccine dose more than six months prior to swabbing). For each variable, we present the point estimate and 95% confidence interval of the Odds Ratio (OR) from the logistic model for vaccination status. The model is parameterised such that ORs greater than 1 indicate a greater probability of being vaccinated. Results are presented in all adults aged 18 years and over (green) and adults aged 50 years and over as well as health care and home care workers (blue).
